# Chest X-ray interpretation does not complement Xpert MTB/RIF in diagnosis of smear-negative pulmonary tuberculosis among TB-HIV co-infected adults in a resource-limited setting

**DOI:** 10.1186/s12879-020-05752-7

**Published:** 2021-01-13

**Authors:** Lydia Nakiyingi, John Mark Bwanika, Willy Ssengooba, Frank Mubiru, Damalie Nakanjako, Moses L. Joloba, Harriet Mayanja-Kizza, Yukari C. Manabe

**Affiliations:** 1 Research Department, Infectious Diseases Institute, Makerere University College of Health Sciences, Kampala, Uganda; 2 Department of Medicine, School of Medicine, Makerere University College of Health Sciences, Kampala, Uganda; 3 Department of Medical Microbiology and Immunology, School of Biomedical Sciences, Makerere University College of Health Sciences, Kampala, Uganda; 4 Johns Hopkins University School of Medicine, Baltimore, MD USA

**Keywords:** Chest X-ray, Resource-limited setting, Tuberculosis, HIV, Diagnosis, Smear-negatives, Uganda

## Abstract

**Background:**

Chest X-ray (CXR) interpretation remains a central component of the current World Health Organization recommendations as an adjuvant test in diagnosis of smear-negative tuberculosis (TB). With its low specificity, high maintenance and operational costs, utility of CXR in diagnosis of smear-negative TB in high HIV/TB burden settings in the Xpert MTB/RIF era remains unpredictable. We evaluated accuracy and additive value of CXR to Xpert MTB/RIF in the diagnosis of TB among HIV-positive smear-negative presumptive TB patients.

**Methods:**

HIV co-infected presumptive TB patients were recruited from the Infectious Diseases Institute outpatient clinic and in-patient medical wards of Mulago Hospital, Uganda. CXR films were reviewed by two independent radiologists using a standardized evaluation form. CXR interpretation with regard to TB was either positive (consistent with TB) or negative (normal or unlikely TB). Sensitivity, specificity and predictive values of CXR and CXR combined with Xpert MTB/RIF for diagnosis of smear-negative TB in HIV-positive patients were calculated using sputum and/or blood mycobacterial culture as reference standard.

**Results:**

Three hundred sixty-six HIV co-infected smear-negative participants (female, 63.4%; hospitalized, 68.3%) had technically interpretable CXR. Median (IQR) age was 32 (28–39) years and CD4 count 112 (23–308) cells/mm^3^. Overall, 22% (81/366) were positive for *Mycobacterium tuberculosis* (Mtb) on culture; 187/366 (51.1%) had CXR interpreted as consistent with TB, of which 55 (29.4%) had culture-confirmed TB. Sensitivity and specificity of CXR interpretation in diagnosis of culture-positive TB were 67.9% (95%CI 56.6–77.8) and 53.7% (95%CI 47.7–59.6) respectively, while Xpert MTB/RIF sensitivity and specificity were 65.4% (95%CI 54.0–75.7) and 95.8% (95%CI 92.8–97.8) respectively. Addition of CXR to Xpert MTB/RIF had overall sensitivity and specificity of 87.7% (95%CI 78.5–93.9) and 51.6% (95%CI 45.6–57.5) respectively; 86.2% (95%CI 75.3–93.5) and 48.1% (95%CI 40.7–55.6) among inpatients and 93.8% (95%CI 69.8–99.8) and 58.0% (95%CI 47.7–67.8) among outpatients respectively.

**Conclusion:**

In this high prevalence TB/HIV setting, CXR interpretation added sensitivity to Xpert MTB/RIF test at the expense of specificity in the diagnosis of culture-positive TB in HIV-positive individuals presenting with TB symptoms and negative smear. CXR interpretation may not add diagnostic value in settings where Xpert MTB/RIF is available as a TB diagnostic tool.

## Background

Tuberculosis (TB) remains a significant public health problem globally and is the main cause of death among persons living with HIV [1]. Key global priorities for TB care and control emphasize improving earlier case detection and linkage to care. Globally, in 2018, a total of 10 million cases of TB were estimated, of which only 6.4 million (64%) were diagnosed and notified to national programs, the remaining 36% of the estimated TB cases were left unreported [1]. These unreported and hence untreated cases are a reservoir of infection with on-going transmission and contribute significantly to the persistently high TB prevalence and mortality particularly among HIV co-infected individuals [1–3]. A recent study showed that in resource-limited settings (RLS), TB accounted for approximately 40% of facility-based HIV/AIDS-related adult deaths, and that in almost half of these, TB was undiagnosed at the time of death [4].

In 2018, Uganda had an estimated TB incidence of 86,000 cases with 24% deaths. In the same year, 34,000 of the new cases were HIV and TB co-infected with an estimated 11,000 HIV-infected individuals having died from TB, making Uganda one of the most affected countries worldwide for TB/HIV co-infection. Of the estimated new TB cases in Uganda, only 65% were notified to the national TB program in 2018 [1, 5]. Delayed and missed TB diagnosis in HIV-positive individuals is largely responsible for the high mortality reported in many RLS endemic for TB/HIV in sub-Saharan Africa (SSA) [6–9].

In many RLS, sputum smear microscopy is still the most widely used up-front diagnostic method at most low-level health centers, and yet it only detects about half of TB cases especially in immunocompromised patients [10]. Low accuracy of sputum smear microscopy has resulted into high frequency of smear-negative TB and increased reliance on empiric decision-making to initiate TB treatment in HIV co-infected patients [11, 12].

In order to achieve the World Health Organization (WHO) goal of ending the TB epidemic by 2035 [13], we need to identify missing TB cases, link them and retain them in appropriate care. One of the strategies to improve identification of these missing cases is improved diagnosis including application of new TB diagnostic tools that have been found to have improved performance. The Xpert MTB/RIF and Ultra assays on the GeneXpert platform (Cepheid, Sunnyvale, CA) have shown the potential to provide rapid, sensitive diagnosis and have been rolled out in several countries in SSA [14, 15]. However, implementation of Xpert MTB/RIF is costly for most low-income countries and requires sophisticated infrastructure that may not be readily accessible in most resource-limited settings [16–18]. Additionally, although the sensitivity of Xpert MTB/RIF assay for pulmonary TB detection was found to be approximately 98% for sputum smear-positive individuals, the sensitivity is much lower at 67–75% for sputum smear-negative HIV-positive individuals [19, 20]. This highlights the need for adjuvant tests and empiric decision-making for smear-negative HIV-positive individuals in particular.

The WHO still considers CXR an important piece in TB diagnosis particularly as an adjuvant test in smear-negative TB diagnostic algorithms [21, 22]. National TB guidelines for most RLS have included use of CXR as part of diagnostic algorithms to complement clinical diagnosis for TB especially for patients with negative sputum smear and/or Xpert MTB/RIF [5, 22]. However, implementation of CXR needs resources to meet operational costs related to electricity, X-ray films, CXR maintenance, and good real-estate facilities that are frequently not available in most RLS. Paucity of personnel that can correctly interpret CXR at most health facilities further challenges CXR implementation [23]. Moreover, the low specificity of CXR is often associated with over-diagnosis of TB, which results in unnecessary TB treatment, wastage of resource and unnecessary toxicity [23–26]. In this Xpert MTB/RIF era, National TB programs in low-income countries need to make important policy decisions on allocation of resources and positioning of CXR within the TB diagnostic algorithms. There is limited published data available on the role of CXR in the Xpert MTB/RIF era to guide policymakers in RLS.

We evaluated the sensitivity, specificity, predictive values and likelihood ratios of CXR interpretation as stand-alone and when in combination with Xpert MTB/RIF, in the diagnosis of smear-negative TB among HIV co-infected patients presenting with symptoms of TB. The goal was to determine the additive value of CXR to smear or Xpert MTB/RIF test in the diagnosis of smear-negative TB among HIV-positive individuals in a high HIV/TB burden setting.

## Methods

### Study design and setting

This study was among participants that were enrolled in a TB diagnostics study that evaluated the lateral flow TB-lipoarabinomannan (TB-LAM) test for the diagnosis of TB among HIV-infected patients with signs and symptoms of TB [27]. The TB diagnostics study recruited outpatients at the adult Infectious Diseases Institute (IDI) Clinic and inpatients at the medical wards of Mulago National Referral Hospital, Kampala Uganda, between January and November 2011.

### Patient recruitment and study population

As previously described [27, 28], the study involved HIV-infected patients at the age of 18 years and above, who were clinically suspected to have active TB (presumptive TB) based on presence of at least one of cough, fever, drenching night sweats or weight loss within the past 4 weeks of enrolment, as reported by the patient. Patients who had taken anti-TB medication for more than 2 days within 60 days prior to enrolment were excluded.

Our previous publications describe the details of the study procedures [27, 28]. In summary, enrolled participants were interviewed for socio-demographic and medical data before study samples were collected. Eligible participants provided two spot sputum samples for conventional TB tests that included; direct fluorescence microscopy (FM) and Ziehl-Neelson (ZN) microscopy; mycobacterial growth indicator tube (MGIT) and Lowenstein-Jensen (LJ) sputum cultures. For participants who were unable to spontaneously expectorate sputum, trained study personnel performed sputum induction. Sputum collection and induction were performed in a separately designated and well-aerated designated area without other patients or staff in the immediate area. The study staff used protective facemasks, gloves and disposable aprons during the entire course of the procedure. Mycobacterial blood cultures and CD4 cell count were performed on blood specimens that were collected at enrolment. Urine TB-LAM testing using a lateral flow assay (TB-LAM Alere, Waltham, MA, USA) was also performed. All male participants and women with a confirmed negative urine pregnancy test had a chest X-ray (CXR) performed.

**Fig. 1:**
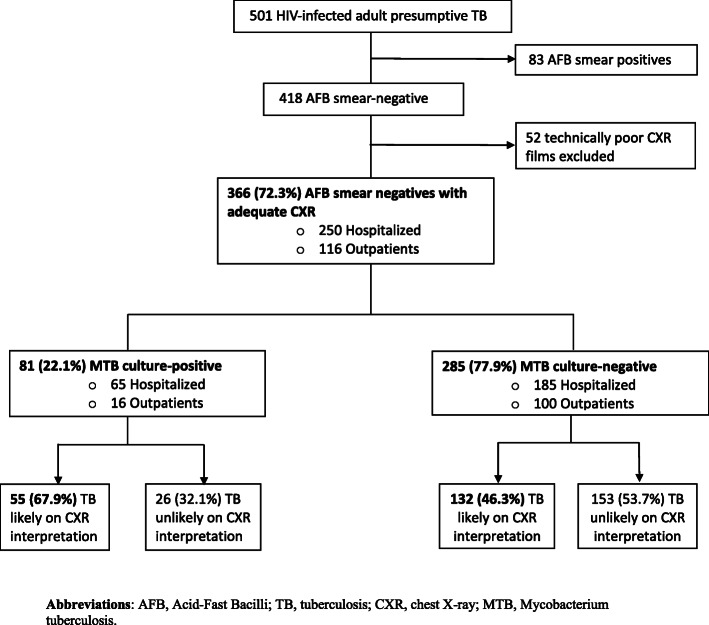
Participant flow chart

Sputum smear-negative participants were those that were smear-negative on both ZN and FM microscopy [21], while MTB culture-positives were those that were MTB positive on any of sputum and/or blood mycobacterial cultures. For this study **(**Fig. 1**),** only sputum smear-negative HIV-infected patients with technically adequate CXR were included. The remaining inclusion and exclusion criteria were consistent with the existing study as described above. Participants without MTB culture results for reference were also excluded from this analysis. Sputum and blood TB cultures were used as reference standard.

### Clinical data

The following general characteristics were collected at enrollment: age, gender, CD4 count (cells/m^3^), sputum smear microscopy, hospital setting, Karnofsky score. In order to assess the participant medical status at the time of visit, antiretroviral (ARV) treatment and co-trimoxazole use, previous TB treatment and previous ARV were obtained. Clinical symptoms that included cough, fever, weight loss, anorexia, excessive night sweats, difficulty in breathing and lymphadenopathy were collected. Cough, fever, weight loss, anorexia and excessive night sweats were retrieved as subjective symptoms that occurred within the past 4 weeks as reported by the patient. All characteristics were ascertained at enrolment.

### CXR radiographs interpretation procedure

Chest X-Rays (CXRs) were performed at enrolment for all study participants except pregnant females. CXR were reviewed by two independent experienced radiologists using a standardized CXR evaluation form. If the two reports differed, a third radiologist was used as a tiebreaker. All readers were blinded to the laboratory data, clinical findings and final diagnosis of the patient. The radiographic features analyzed were infiltrates (upper/mid/lower), fibrosis, cavity, miliary disease, adenopathy, pleural effusion, pleural thickening and others. Based on the general impression of the radiologist, CXR interpretation with regard to TB was reported as either positive (consistent with TB) or negative (normal or unlikely TB) as the main outcome.

### Laboratory procedures

To identify sputum smear-negative participants, smear microscopy was performed on two spot sputum samples, using FM and ZN. Participants were classified as sputum smear-negative if they were smear-negative on both ZN and FM microscopy. The same sputum samples were used to perform MGIT and LJ cultures. Each participant in addition had a mycobacterial blood culture. A positive MTB culture was defined as presence of at least one positive TB culture on any of the three TB cultures MGIT, LJ or MTB blood culture. Details of the laboratory procedures for all study tests have been described earlier [27, 28]. All confirmed MTB positive patients by any of the TB tests performed during the study were promptly initiated on TB treatment by the attending clinician according to the Uganda Ministry of Health TB and Leprosy program guidelines [5].

### Statistical analysis

The analysis was performed on data from a subset of participants enrolled in a TB diagnostic accuracy study of the lateral flow TB-lipoarabinomannan (TB-LAM) test for the diagnosis of TB among HIV-infected presumptive TB patients. All statistical analyses were performed using Stata version 12.0. Categorical data were summarised using frequencies, proportions and percentages. Continuous data were summarised using means and median. Accuracy (sensitivity, specificity, positive and negative predictive values as well as likelihood ratio positive and negative) of CXR interpretation and when combined with Xpert MTB/RIF in the diagnosis of smear-negative TB was calculated using sputum and/or blood TB cultures as reference standard. All statistics were two-tailed and *p*-values less than 0.05 were considered statistically significant.

## Results

### Baseline characteristics

Of the 501 HIV-infected presumptive TB patients who provided sputum samples for microscopy, 418 (83.4%) were sputum smear-negative. Of the 418 smear-negatives, 366 with MTB culture results available had technically adequate chest radiographs and thus were included in this analysis **(**Fig. 1**)**. Of the 366 sputum smear-negative participants eligible for analysis, 68.3% (250/366) were inpatients, 63.4% (232/366) were female; median age was 32 (IQR 28, 39) years and median CD4 count was 112 (IQR 23–308) cells/mm^3^.

### MTB culture results

Of the 366 sputum smear-negative participants analyzed, 81/366 (22.1%) were positive for *Mycobacterium tuberculosis* (MTB) complex on blood and/or sputum TB cultures; of which, 48/81 (59.3%) were MTB culture positive on sputum alone, 28/81 (34.6%) were positive on both sputum and blood cultures while 5/81 (6.2%) were positive on blood culture alone. Majority of the MTB culture-positives were inpatients (65/81, 80.2%). The median CD4 cell count was significantly lower in the MTB culture-positive compared to the culture-negative participants [79 (IQR 15–179) versus 130 (IQR 26–331) cells/mm^3^, *p*=0.011 respectively]. A comparison of clinical and laboratory characteristics between MTB culture-positive and negative participants is shown in Table 1.

**Table 1:** Characteristics of HIV-positive, smear-negative presumptive participants, comparing MTB culture-positives and negatives (*n*=366)

Participants characteristics	Overall (***N***=366)	Culture positives (***N***=81)	Culture negatives (***N***=285)	***P***-value
**Clinical characteristics**				
Age at visit time (Median (IQR)	32 (28, 39)	31 (27, 37)	33 (28, 40)	0.064
CD4 count (cells/mm3) (Median, IQR)	112 (23, 308)	79 (15, 179)	130 (26, 331)	**0.011**
Gender: Female (n, %)	232 (63.4)	43 (53.1)	189 (66.3)	**0.029**
Inpatient participants	250 (68.3)	65 (80.3)	185 (64.9)	**0.009**
Cough Present	359 (98.1)	80 (98.8)	279 (97.9)	1.000
Fever present	333 (91.0)	75 (92.6)	258 (90.5)	0.665
Weight loss present	325 (88.8)	80 (98.8)	245 (86.0)	**0.001**
**Medication history**				
Antiretroviral therapy (n, %)	137 (37.4)	24 (29.6)	113 (39.6)	**0.037**
Cotrimoxazole Prophylaxis	341 (93.2)	78 (96.3)	263 (92.3)	0.299
Previous medication for TB disease	67 (18.3)	9 (11.1)	58 (20.4)	**0.032**
**TB tests results**				
Xpert MTB/RIF positive	65 (17.8)	53 (65.4)	12 (4.2)	**0.000**
Urine TB LAM Positive (n, %)	50 (13.7)	28 (34.6)	22 (7.7)	**0.000**
CXR interpreted as TB (n, %)	187 (51.1)	55 (67.9)	132 (46.3)	**0.001**
**Radiological Characteristics** (n, %)				
Upper infiltrates^a^	143 (39.1)	52 (64.2)	91 (31.9)	**0.000**
Lower Infiltrates	150 (41.0)	46 (56.8)	104 (36.5)	**0.001**
Cavity	15 (4.1)	2 (2.5)	13 (4.6)	0.537
Miliary disease	6 (1.6)	4 (4.9)	2 (0.7)	**0.023**
Pleural Effusion	39 (10.7)	13 (16.0)	26 (9.1)	0.100
Pleural Thickening	35 (9.6)	12 (14.8)	23 (8.1)	0.085
Adenopathy	13 (3.6)	5 (6.2)	8 (2.8)	0.172
Fibrosis	20 (5.5)	4 (4.9)	16 (5.6)	1.000

**Abbreviations**: *TB* Tuberculosis; *ART* Antiretroviral therapy; *CXR* Chest X-ray; *LAM* Lipoarabinomannan; *IQR* Inter-quartile range; ^a^ means infiltrates in upper or middle lobe

### Accuracy of chest X-ray interpretation

Overall, of the 366 participants with interpretable CXR, 187/366 (51.1%) had CXR interpreted as consistent with pulmonary TB, of which only 55/187 (29.4%) had MTB culture confirmed TB on sputum and/or blood (Table 1). Abnormal CXR findings interpreted as TB were more frequent among the MTB culture positive compared to MTB culture-negative smear-negative HIV participants (67.9% versus 46.3% *p*=0.001). The most frequent abnormalities reported include; lower lobe infiltrates (41%), upper and middle lobe infiltrates (39.1%), and pleural disease including pleural effusion, 10.7% and pleural thickening, 9.6%. All these CXR abnormalities were more common among MTB culture positives (Table 1). Miliary TB (1.6%) and cavities (4.1%) were rare among the participants and majority (4 out of 6) of the miliary TB participants on CXR also had positive MTB cultures but negative urine TB-LAM.

The sensitivity and specificity of CXR interpretation determined using MTB culture as reference standard in the diagnosis of smear-negative TB in HIV-co infected individuals were 67.9% (95% CI 56.6–77.8%) and 53.7% (95% CI 47.7–59.6%) respectively (Table 2). The positive and negative predictive values were 29.4% (95% CI 23–36.5%) and 85.5% (95% CI 79.4–90.3%) respectively.

**Table 2:** Accuracy of CXR interpretation and when combined with Xpert MTB/RIF in HIV-related smear-negative TB diagnosis

Setting	Accuracy Index	CXR Interpretation	Xpert MTB/RIF test	CXR plus Xpert MTB/RIF test
Overall	Sensitivity (95% CI)	67.9% (56.6–77.8)	65.4% (54.0–75.7)	87.7% (78.5–93.9)
	Specificity (95%CI)	53.7% (47.7–59.6)	95.8% (92.8–97.8)	51.6% (45.6–57.5)
	PPV (95% CI)	29.4% (23.0–36.5)	81.5% (70.0–90.1)	34.0% (27.6–40.8)
	NPV (95% CI)	85.5% (79.4–90.3)	90.7% (86.8–93.7)	93.6% (88.6–96.9)
	Likelihood ratio (+)	1.47 (1.21–1.78)	15.54 (8.74–27.64)	1.81 (1.57–2.09)
	Likelihood ratio (−)	0.60 (0.43–0.84)	0.36 (0.27–0.49)	0.24 (0.13–0.43)
	Area under ROC curve	0.61 (0.55–0.67)	0.81 (0.75–0.86)	0.70 (0.65–0.74)
Inpatients	Sensitivity	66.2% (53.4–77.4)	64.6% (51.8–76.1)	86.2% (75.3–93.5)
	Specificity	50.3% (42.8–57.7)	95.7% (91.7–98.1)	48.1% (40.7–55.6)
	PPV (95% CI)	31.9% (24.1–40.4)	84.0% (70.9–92.8)	36.8% (29.2–45.0)
	NPV (95% CI)	80.9% (72.5–87.6)	88.5% (83.2–92.6)	90.8% (83.3–95.7)
	Likelihood ratio (+)	1.33 (1.06–1.67)	14.94 (7.41–30.13)	1.66 (1.40–1.97)
	Likelihood ratio (−)	0.67 (0.47–0.97)	0.37 (0.27–0.51)	0.29 (0.15–0.54)
	Area under ROC curve	0.58 (0.51–0.65)	0.80 (0.74–0.86)	0.67 (0.62–0.73)
Outpatient	Sensitivity	75.0% (47.6–92.7)	68.8% (41.3–89.0)	93.8% (69.8–99.8)
	Specificity	60.0% (49.7–69.7)	96.0% (90.1–98.9)	58.0% (47.7–67.8)
	PPV (95% CI)	23.1% (12.5–36.8)	73.3% (44.9–92.2)	26.3% (15.5–39.7)
	NPV (95% CI)	93.8% (84.8–98.3)	95.0%(88.8–98.4)	98.3% (90.9–100)
	Likelihood ratio (+)	1.88 (1.29–2.72	17.19 (6.23–47.45)	2.23 (1.72–2.90)
	Likelihood ratio (−)	0.42 (0.18–0.99)	0.33 (0.16–0.67)	0.11 (0.02–0.72)
	Area under ROC curve	0.67 (0.56–0.79)	0.82 (0.70–0.94)	0.76 (0.68–0.84)

**Abbreviations**: *CI* Confidence Intervals; *CXR* Chest X-ray; *PPV* Positive predictive value; *NPV* Negative predictive value; (+),Positive; (−),Negative; *ROC* Receiver Operating Characteristic curve

Table 2 shows accuracy of CXR interpretation and when combined with Xpert MTB/RIF for the diagnosis of smear-negative TB in HIV using Mycobacterial cultures as reference standard.

Restricting the analysis to inpatients, 135/250 (54%) had CXR interpreted as consistent with TB, of which 43/135 (31.9%) were MTB culture positive. The sensitivity and specificity of CXR interpretation among inpatient participants were therefore 66.2% (95%CI 53.4–77.4%) and 50.3% (95% CI 42.8–57.7%) respectively. Analysis among the outpatients alone found, of the 116 outpatients, 52 (54%) had CXR interpreted as consistent with TB, of which 12/52 (23.1%) were MTB culture positive. The sensitivity and specificity of CXR interpretation among the outpatient participants were 75% (95%CI 47.6–92.7%) and 60% (95% CI 49.7–69.7%) respectively (Table 2).

### Accuracy of Xpert MTB/RIF test

Xpert MTB/RIF test was positive in 65 (17.8%) of the study participants; 53 (65.4%) were among the 81 MTB culture positives and 12 (4.2%) among the 285 MTB culture negatives. Therefore, for the diagnosis of smear-negative TB among HIV co-infected participants, Xpert MTB/RIF test had overall sensitivity and specificity of 65.4% (95%CI 54.0–75.7%) and 95.8% (95%CI 92.8–97.8%) respectively (Table 2). Among inpatients only, Xpert MTB/RIF sensitivity and specificity were 64.6% (95%CI 51.8–76.1%) and 95.7% (95%CI 91.7–98.1%) respectively while among outpatients, the sensitivity and specificity were 68.8% (95%CI 41.3–89%) and 96% (95%CI 90.1–98.9%) respectively (Table 2).

### Incremental accuracy of CXR to Xpert MTB/RIF

Addition of CXR interpretation to Xpert MTB/RIF test diagnosed 209 (57.1%) of the 366 HIV-infected smear-negatives as pulmonary TB, of which 71/209 (34%) participants had MTB culture confirmed TB. Using MTB culture as reference standard, the sensitivity of Xpert MTB/RIF when CXR interpretation was added for the diagnosis of TB among smear-negative HIV-infected patients was 87.7% (95%CI 78.5–93.9%) and the specificity was 51.6% (95%CI 45.6–57.5%). The positive and negative predictive values were 34% (95% CI 27.6–40.8%) and 93.6% (95% CI 88.6–96.9%) respectively.

Among inpatients, the sensitivity and specificity of Xpert MTB/RIF when CXR interpretation was added for the diagnosis of culture confirmed TB among HIV-infected smear-negatives were 86.2% (95%CI 75.3–93.5%) and 48.1% (95%CI 40.7–55.6%) respectively, while among outpatients, the sensitivity and specificity were 93.8% (95%CI 69.8–99.8%) and 58% (95% CI 47.7–67.8%) respectively (Table 2).

Further analysis involving only TB treatment naïve patients did not show any improvement in the performance of CXR in the diagnosis of PTB among TB treatment naïve smear-negative HIV patients (sensitivity 67.6, 95% CI 55.7–78.0%; specificity 59.6, 95% CI 52.8–66.0%). Additionally, compared to the overall participants, no significant difference in incremental yield was observed when CXR was used in combination with Xpert MTB/Rif in the population of TB treatment naïve patients (sensitivity 86.5 95%CI 76.5–93.3% and specificity 57.3 95% CI 50.6–63.9%) (Table 3).

**Table 3:** Accuracy of CXR and CXR plus GeneXpert in smear-negative TB diagnosis among TB-treatment naïve participants

Accuracy index	CXR Interpretation	CXR plus Xpert MTB/RIF test
Sensitivity (95% CI)	67.6% (55.7–78)	86.5% (76.5–93.3)
Specificity (95% CI)	59.6% (52.8–66)	57.3% (50.6–63.9)
PPV (95% CI)	35.5% (27.6–44)	40% (32.3–48)
NPV (95% CI)	84.8% (78.2–90)	92.8% (87.2–96.5)
Likelihood ratio (+)	1.67 (1.34–2.09)	2.03 (1.7–2.42)
Likelihood ratio (−)	0.545 (0.385–0.77)	0.236 (0.131–0.424)
AUC (ROC area)	0.636 (0.573–0.698)	0.719 (0.668–0.77)

*CI* Confidence Intervals; *CXR* Chest X-ray; *PPV* Positive predictive value; *NPV* Negative predictive value; (+),Positive; (−), Negative; *ROC* Receiver Operating Characteristic curve

Table 3 shows accuracy of CXR interpretation and when CXR is combined with Xpert MTB/RIF for the diagnosis of smear-negative TB among TB treatment naive HIV-positive patients using Mycobacterial cultures as reference standard.

## Discussion

Chest X-Ray (CXR) remains a useful adjuvant test in smear-negative pulmonary TB to guide further patient management in high TB/HIV prevalence settings. Given the recent widespread roll-out of Xpert MTB/RIF in most low-income countries, we evaluated the role of CXR in the diagnosis of smear-negative TB among HIV-positive presumptive TB patients in the Xpert MTB/RIF era. Overall, we found poor performance of CXR interpretation in the diagnosis of culture- positive smear-negative TB among HIV-positive participants. We found that, although CXR increased sensitivity of Xpert MTB/RIF assay, this was at the expense of specificity. Given its high negative predictive value, a normal CXR could reliably exclude TB in HIV-positives with negative sputum smear, however, the applicability of CXR in settings with Xpert MTB/Rif is limited by the challenges of CXR application faced in most RLS. Our study further found that combining CXR with Xpert MTB/RIF reduced the specificity of Xpert MTB/RIF in the diagnosis of smear-negative TB. This implies that use of CXR as an adjuvant to Xpert MTB/RIF results into inaccurate diagnosis of TB among HIV co-infected presumptive TB patients with negative smear.

Our findings corroborate with earlier reports from high prevalence TB/HIV settings in SSA which also found poor performance, particularly low specificity, of CXR interpretation in the diagnosis of culture-confirmed TB among smear-negative HIV co-infected individuals [24, 26, 29–31].

Poor performance of CXR in TB diagnosis in HIV co-infected individuals is often attributed to the non-specific CXR patterns and difficulty in interpretation [23, 25]. Results of a chest radiograph often depend on the severity and presentation of TB, which all depend on the stage of the HIV disease, with advanced HIV disease more likely to present with non-specific patterns compared to early-stage HIV disease. Moreover, CXR findings can be normal in up to 25–50% of patients with culture-confirmed TB [23, 25, 26]. Our study population comprised HIV co-infected individuals, majority of whom had advanced HIV disease (median CD4 count 112 cells/mm^3^) and this could potentially explain the non-specific patterns inaccurately interpreted as TB, and thus the low specificity seen in our study.

CXR interpretation in HIV co-infection is further complicated by the presence of other HIV-related pulmonary diseases that could radiologically mimic TB; commonly, bacterial pneumonia [23, 24]. Several studies from SSA [24, 26, 30] have reported presence of other HIV-related pulmonary conditions that radiologically mimic TB that have led to misinterpretation of chest radiographs as TB. This too could explain the poor CXR performance in this study that was performed among entirely HIV-positive individuals. However, our study did not evaluate other possible non-TB pulmonary conditions that could explain the inaccurate CXR interpretation.

Poor performance of CXR interpretation in this study could also be explained by poor quality of CXR films, which in turn is dependent on the skill of the technician, and the quality of the CXR equipment used. Studies have shown accuracy of CXR interpretation is dependent on the quality of the CXR film [23, 30]. In most peripheral health centres in RLS where CXR services are available, the quality of the radiographs produced from the poor quality CXR equipment is often not good enough to improve interpretation [23]. To improve accuracy of CXR interpretation in our study, CXR interpretation was performed by expert radiologists.

Our study further found that CXR interpretation did not complement Xpert MTB/RIF diagnostic performance in smear-negative TB diagnosis in this low-income country with high TB/HIV prevalence. Instead, we noted decreased accuracy with loss in specificity of Xpert MTB/RIF test when additively used with CXR interpretation i.e. Xpert MTB/RIF specificity dropped from 95.8% when used as stand-alone test to 51.6% when additively used with CXR interpretation. A similar trend was seen when results were stratified by setting (outpatients versus inpatients). Our findings are similar to an earlier report, which also found a reduction in specificity of Xpert MTB/RIF when additively used with CXR interpretation [31]. The reduction in specificity is attributable to the non-specific nature of CXR patterns in HIV disease, which results into inaccurate interpretation [23, 26]. These findings imply that use of CXR interpretation as an additive test to Xpert MTB/RIF test in centers where Xpert MTB/RIF is available as a TB diagnostic increases the likelihood of inaccurate diagnosis of TB, which consequently puts more financial strain to TB programs in RLS and leads to misuse of already scarce resources. However, based on our recent findings from the same patient population, CXR could complement urine TB Lipoarabinomannan (LAM) if Xpert MTB/RIF is not available [28]. The previous findings showed that an algorithm in which CXR is performed following urine TB LAM test significantly improves diagnosis of TB among smear-negative HIV-positive patients with a high negative predictive value, especially when CD4 counts are < 100cells/mm^3^ [28]. This was not the case when CXR was combined with Xpert MTB/RIF test in the diagnosis of TB in smear-negative HIV-positive patients.

Of note, our study found lower lung field infiltrates (41%) and pleural disease among the most frequent abnormalities reported by the expert radiologist. This is similar to earlier findings from other studies on CXR features among HIV co-infected individuals [23–26, 30]. These atypical CXR features are not surprising in our study, which included entirely HIV co-infected patients, many of whom had immunologically advanced HIV disease. Advanced HIV disease could also explain the miliary TB disease CXR pattern seen in a few of our study patients. Cavities were rare among our study participants as is expected in HIV-positive patients with advanced disease [23, 25].

This study had strengths. First, our study included both sputum and blood MTB culture as the standard of reference, which improved the strength of our reference standard for TB diagnosis. Inadequacy of gold standard is often a limitation in TB diagnostics evaluations. Second, in order to improve CXR interpretation, we used two expert radiologists to interpret the chest radiographs, and in case of variability, a third radiologist was used as a tiebreaker. Third, our study population and setting well represent a real-world situation that is likely to be found in high prevalence HIV/TB settings in low-income countries. We studied a population of HIV-infected individuals with an increased risk of morbidity and mortality from TB that is targeted in the End TB strategy. Lastly, we used a relatively simple CXR evaluation form in the interpretation of the CXR, which is a more practical approach since hospitals in RLS often lack personnel that can interpret CXR with expertise and are often understaffed. By using a simple and easy-to-use CXR interpretation form, we assigned the accuracy of the CXR in this study more agreeable with resource-constrained settings. However, the study results cannot be generalized to populations that were not included in this study, particularly pregnant women and children.

## Conclusion and recommendation

In this high HIV/TB burden resource-limited setting, CXR interpretation had low diagnostic utility in HIV co-infected patients presenting with TB symptoms and negative smear. Addition of CXR to Xpert MTB/RIF increased sensitivity but reduced specificity in the diagnosis of smear-negative TB among HIV-positive patients. CXR interpretation may not add diagnostic value in settings where Xpert MTB/RIF is available as a TB diagnostic tool.

## Data Availability

The datasets used and/or analyzed during the current study are available from the corresponding author on reasonable request.

## Abbreviations

CI: Confidence interval
CXR: Chest x-ray
FM: Fluorescence microscopy
IRB: Institutional review board
LJ: Lowenstein-jensen
MGIT: Mycobacterial growth indicator tube
MTB: *Mycobacterium tuberculosis*
TB: Tuberculosis
TB-LAM: TB-lipoarabinomannan
ZN: Ziehl-neelson
